# Observation of Alectinib‐ and Crizotinib‐ included chemotherapy in children with ALK‐positive anaplastic large cell lymphoma: A single institutional experience

**DOI:** 10.1002/cam4.5479

**Published:** 2022-11-21

**Authors:** Yingyi He, Kunlin Pei, Hui Zhang, Jiayi Wang, Xiaoling Su, Wenting Gan, Pengfei Wang

**Affiliations:** ^1^ Department of Pediatric Hematology & Oncology Guangzhou Women and Children's Medical Center Guangzhou Guangdong China; ^2^ Guangzhou Medical University Guangzhou Guangdong China

**Keywords:** ALK inhibitor, ALK‐positive, anaplastic large cell lymphoma, childhood, outcome

## Abstract

Approximately one‐third children with anaplastic large cell lymphoma (ALCL) relapse after completion of chemotherapy, particularly for those high‐risk patients. The introduction of novel therapeutic modalities is much needed for these sub‐group patients. Two groups (*n* = 3, *n* = 4) of ALCL patients were treated with crizotinib‐ and alectinib‐included ALCL‐99 therapy, respectively, achieving complete remission rates of 66.7% and 100%. Two patients of crizotinib group relapsed, while none relapsed among the alectinib‐treated patients. Adding alectinib instead of crizotinib sufficiently suppressed and maintained the deep *NPM*‐*ALK* molecular response. ALK inhibitors were well tolerated with only grade 1 adverse events in both groups. Though a relatively small case number, this study raised the possibility that alectinib‐included therapeutic regimens may benefit the early response, in‐depth molecular remission, and persistent remission to some extent. Further studies are warranted to validate our preliminary findings.

## Graphical abstract

Our study raised the possibility that alectinib‐included therapeutic regimens might benefit the early response, in‐depth molecular remission, and persistent remission in children with ALK‐positive Anaplastic large cell lymphoma.

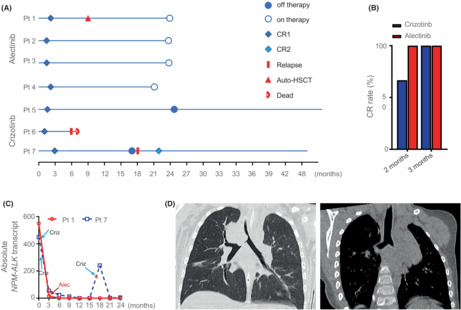

Anaplastic large cell lymphoma (ALCL) accounts for 10% ‐ 15% of all pediatric non‐Hodgkin lymphoma (NHL).^1^, ^2^ The well‐established standard first‐line ALCL therapies include intensive T‐cell malignancies strategy and B‐NHL analogous therapies. The event‐free survival (EFS) rate of childhood ALCL is ~70%.^3^, ^4^, ^5^, ^6^, ^7^, ^8^, ^9^, ^10^ Unfortunately, approximately one‐third experience disease recurrence, with half of them eventually succumbing to the disease.^11^, ^12^ Although autologous stem cell transplant (auto‐SCT) represents an optional salvage therapy for the recurrent cases, not all patients could tolerate this treatment or achieve a sufficiently good response to the high‐dose chemotherapy.^13^ Thus, novel agents are urgently needed for preventing the recurrence.

Unlike adult ALCL, the majority of pediatric cases (>95%) demonstrated overexpression of anaplastic lymphoma kinase (ALK).^14^ Constitutive ALK activation due to rearrangements between *NPM* and *ALK* contributes to the ALCL tumorigenesis.^15^ All this evidence points to the therapeutic potential of ALK inhibitor in ALCL. Crizotinib was the first ALK inhibitor to be approved for use in patients with ALK‐rearranged non‐small cell lung cancer (NSCLC).^16^ For pediatric ALCL, crizotinib demonstrated efficacy as a single agent in pediatric patients with relapsed ALCL with an objective response rate of 88% and a complete remission rate of 81% in the Children's Oncology Group trial (ADVL0912 trail).^17^, ^18^ As evidence by these results of the trial, the U.S. Food and Drug Administration has approved crizotinib for the treatment of children and young adults with relapsed/refractory (R/R) ALK‐rearranged ALCL. Though crizotinib confers remarkable clinical outcomes in ALK‐rearranged NSCLC, progression invariably occurs. Retrospective studies have shown that patients treating with crizotinib experience relapse within 2 years of treatment or often develop central nervous system (CNS) metastases.^19^, ^20^ Thus there is an unmet need for a new ALK inhibitor that could overcome crizotinib resistance and reach adequate CNS concentration.^19^, ^20^

In this regard, a number of new ALK inhibitors are currently under development. Preclinical and clinical results have shown that these next‐generation ALK inhibitors can target at least one or more of the ALK mutants resistant to crizotinib. Meanwhile, these novel ALK inhibitors have shown higher activity against intracranial tumors,^21^ among which, alectinib is a potent and selective tyrosine kinase inhibitor(TKI) of ALK and can partially rescue resistance incurred by crizotinib‐related ALK mutation. Impressively, in a well‐designed phase III study, alectinib represented significantly superior systemic and CNS efficacy over crizotinib in treating newly‐diagnosed ALK‐positive NSCLC and ALK‐positive NSCLC with crizotinib treatment in prior.^22^, ^23^ However, evidence of the frontline ALK inhibitors in ALK‐positive ALCL is minimal, even less in pediatric cases.

In this retrospective study, we reported our preliminary findings of alectinib and crizotinib in treating children with high‐risk ALCL. The alectinib‐included treatment for high‐risk ALCL study was approved by the institutional review board (IRB) at Guangzhou Women and Children's Medical Centre (2020–136). Meanwhile, crizotinib was prescribed for those three patients upon physician's experience and decision. Informed consent was provided by the patients' legal guardians and/or patients themselves if they were over 8 years old. Seven patients who met the inclusion criteria were included into this retrospective study (Table 1). The patient median age was 4.7 years (range, 1–10.4), with five males and two females. Of these seven patients, three patients received crizotinib‐ and four patients received alectinib‐included ALCL‐99 therapy (Table 1 and Data S1). All these seven ALCL patients well responded to the ALK inhibitor‐included regimens and achieved CR in the first 3 months (Table 1 and Figure 1A). As shown in Figure 1B, the CR rate after two cycles of chemotherapy was 66.7% (two out of three patients) and 100% in crizotinib‐ and alectinib‐included regimens respectively. At the time of three cycles of chemotherapy, no difference was observed among these two groups. Regarding the adverse events (Table 1), only patient 1 in the alectinib group experienced grade 1 erythema multiforme, and patient 5 in the crizotinib group experienced grade 1 oral mucositis. At the time of submission, we had completed the last follow‐up for all patients via telephone. As illustrated in Table 1 and Figure 1A, the follow‐up duration varied from 6 to 61 months (patient 1, 28 months; patient 2, 26 months; patient 3, 26 months; and patient 4, 24 months; patient 5, 61 months; patient 6, 6 months; patient 7, 46 months). In the alectinib group, all four patients remained healthy without relapse or progression event up to now during the follow up varying from 24 to 28 months. In the crizotinib group, the crizotinib monotherapy was terminated in patient 5 after 30 cycles of crizotinib's treatment. Luckily, he was still in healthy condition at the time of submission, achieving up to 61 months' CR. Patient 6 experienced early relapse at 6 months from diagnosis during the crizotinib monotherapy. Thus, crizotinib was discontinued for this patient and finally she died of disease progression one month later. Patient 7 responded well to crizotinib‐included ALCL‐99 treatment within 8 weeks of treatment, and he received a total of 17 months' crizotinib therapy. However, he experienced his first relapse one month after crizotinib discontinuation. Due to social economic status and well crizotinib tolerability, his parents chose crizotinib monotherapy as his salvage therapy. A second CR was achieved after six cycles of crizotinib treatment when the second relapse recurred. Of these two relapsed patients, the median relapse time was 11.5 months (ranging from 6 to 17 months). The median follow‐up duration of these two surviving patients was 53 months (ranging from 46 to 61 months) after receiving crizotinib.

**TABLE 1:** Clinical characteristics, treatment response, adverse events and follow up of 7 ALK inhibitor‐ treated pediatric patients with newly‐diagnosed ALCL

Patients/Group	Alectinib (*N* = 4)				Crizotinib (*N* = 3)		
	Patient 1	Patient 2	Patient 3	Patient 4	Patient 5	Patient 6	Patient 7
Age (y)	1.3 y	1 y	3 y	8 y	5 y	4.5 y	10.4 y
Sex	Female	Male	Male	Male	Male	Female	Male
Stage (IPNHLSS)	Stage IV^a^	Stage III^b^	Stage III^c^	Stage III^d^	Stage III^e^	Stage IV^f^	Stage IV^g^
ALK method	IHC, PCR	IHC	IHC	IHC	IHC	IHC	IHC, PCR
ALK fusion	NPM	NPM	NPM	N.D	N.D	N.D	NPM
B symptoms	Yes	Yes	Yes	Yes	Yes	Yes	Yes
HLH	Yes	No	Yes	Yes	No	No	Yes
Histological subtypes	Small cell	Common	Small cell	Common	Common	Common	Common
Bronchus involvement	No	No	RMB	No	No	No	No
ALK inhibitor dose	300 mg/d	300 mg/d	300 mg/d	300 mg/d	250 mg/d	250 mg/d	500 mg/d
Best response to ALK inhibitor	CR	CR	CR	CR	CR	CR	CR
Time to first CR/PR	8 weeks	6 weeks	6 weeks	8 weeks	6 weeks	12 weeks	4 weeks
ALK inhibitor duration/ discontinuation	24 mo^h^/No	24 mo/Yes	24 mo/Yes	23 mo/No	24 mo/Yes	6 mo/Yes	17 mo/Yes
Event							
Relapse	No	No	No	No	No	Yes^i^	Yes^j^
Death	No	No	No	No	No	Yes	No
Duration of best ALK inhibitor response	≥24 mo	≥24 mo	≥24 mo	≥23 mo	≥60 mo	3 mo	18 mo
Proceed to Auto‐SCT	Yes	No	No	No	No	No	No
Current status	CR	CR	CR	CR	CR	death	Relapse
Follow‐up since ALK inhibitor introduction (months)	28 mo	26 mo	26 mo	23 mo	61 mo	6 mo	46 mo
Adverse events							
Skin rash	No	Yes	No	No	No	No	No
Extra skin	No	No	No	No	Yes	No	No
ALK inhibitor dose modification	Not required	Not required	Not required	Not required	Not required	Not required	Not required

Abbreviations: IHC, immunohistochemistry; N.D, not done; NPM, nucleophosmin; PCR, polymerase chain reaction; RMB, right main bronchus.

^a^ Patient 1 was diagnosed with stage IV disease involving cervical/celiac nodal disease plus liver, spleen, bone marrow, and skin.

^b^ Patient 2 was diagnosed with stage III disease with mediastinal tumor, bronchial involvement, and multiple lymphomatous involvement of cervical, supraclavicular, mediastinal, axillary, prominent right hilar lymph node, as well as pleural dissemination.

^c^ Patient 3 was diagnosed with stage III disease involving intrathoracic tumor (mediastinal, bronchial, lung, pericardium, and pleura), and skin.

^d^ Patient 4 was diagnosed with stage III disease involving intrathoracic tumor (lung, pericardium, and pleura), and multiple lymph node enlargements involving hilar, mediastinal, perihepatic and retroperitoneal lymph nodes.

^e^ Patient 5 was diagnosed with stage III disease with intra‐abdominal disease (kidney) and soft tissues.

^f^ Patient 6 was diagnosed with stage IV disease involving multiple lymph node enlargements from the cervical to inguinal areas plus liver, spleen, and bone marrow.

^g^ Patient 7 was diagnosed with stage IV disease involving cervical/axillary nodal disease plus hepatomegaly, splenomegaly, bone marrow and skin.

^h^ Patient 1 shifted from crizotinib to alectinib from Feb 2020, considering her bone marrow showed a persistent positivity of the NPM‐ALK fusion transcript and TCR arrangement, which were highly suspicious for disease relapse.

^i^ Patient 6 relapsed after cycle 8 of crizotinib therapy.

^j^ Patient 7 Relapsed after one month of crizotinib discontinuation.

**FIGURE 1:**
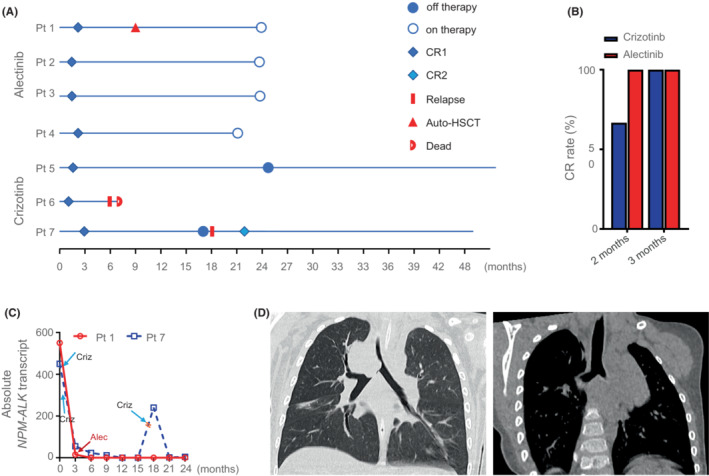
Treatment response characteristics in children with ALK‐positive ALCL treated with Alectinib or Crizotinib. (A) A diagram of treatment response and follow up of children with ALCL in this study (dark blue diamond: first complete remission; light blue diamond: second complete remission; blue circle: on therapy; solid red bar: relapse; red triangle: auto‐HSCT; red half circle: dead; solid blue circle: off therapy). (B) The overall response of ALCL to alectinib‐ and crizotinib‐ included therapy was evaluated at 2 and 3 months after treatment. (C) The levels of NPM‐ALK transcript of patient 1 and 7 were measured by quantitative PCR. The red line and dash blue line represent patient 1 and 7 respectively. The light blue arrow and dark red arrow represent crizotinib and alectinib treatment respectively. The orange parallel lines represent the discontinuation of crizotinib in patient 1. (D) Representative CT scan images of patient 3 before (left panel) and after (right panel) alectinib‐included ALCL therapy.

Persistence of *ALK*‐fusion transcripts negatively affects the prognosis,^24^, ^25^ levels of *NPM*‐*ALK* transcripts were quantified using samples from two BM involvement patients during ALK inhibitor treatment. As shown in Figure 1C, the *NPM*‐*ALK* transcript in patient 7 was undetectable until four months after eleven months of crizotinib exposure, and thus he finally relapsed unfortunately. Interestingly, patient 1 was initially treated with crizotinib‐included chemotherapy and achieved CR after two cycles of therapy. Of note, the *NPM*‐*ALK* transcript was also detectable. Upon the physician's decision, alectinib was promptly introduced and the *NPM‐ALK* transcript quickly became undetectable within three months of alectinib therapy and remained undetectable up to now.

In this report, patient 3 presented with respiratory distress, pointing to bronchial involvement that was sporadically reported in ALCL patients, particularly in young patients. CT scan (Figure 1D and Figure S2) revealed a large right‐middle mediastinal mass measures 3.2 × 2.8 × 2.2 cm, located into right principal bronchus and extended to the trachea. Obstruction of near 95% of the principal bronchus and the right principal bronchus was identified under bronchoscopy. Using thoracoscopy, the mediastinal mass and superior lobe of the right lung were successfully resected. Alectinib‐included therapy was administered immediately after the diagnosis, and CR was achieved after 6 weeks' therapy (Figure 1D and Figure S2). After six cycles of alectinib‐included ALCL‐99 therapy, this patient remained in CR without evidence of recurrence based on physical examinations and CT scan evaluation at the time of the submission.

Though a variety of treatment strategies have been proposed for treating children with ALCL In the last three decades, an average of ~60%cure rates were observed,^26^ suggesting that current chemotherapy has hit a plateau at treating childhood ALCL. Thus, introducing novel therapeutic modalities is highly necessary for successfully managing children with ALCL in up‐front settings. ALK rearrangement is a hallmark of pediatric ALCL, suggesting the therapeutic potential of ALK inhibitor in treating ALCL. Indeed, several reports have found that crizotinib was highly effective against ALK‐rearranged ALCL or solid tumors.^17^, ^18^, ^27^ However, relapse was recorded in childhood as well as adulthood ALK‐rearranged ALCL patients who receiving crizotinib‐included therapy.^28^ All these evidences have pointed to that crizotinib appears to fail to prevent relapse, even though adding crizotinib leads to relative higher ORR.^18^ Plausible reasons might be the delayed crizotinib introduction or the inherent deficiency of crizotinib in relapse prevention. However, relapse at early stage was also observed in two patients (patient 6 and 7) receiving crizotinib‐included chemotherapy in our study, excluding the delayed introduction as a reason for relapse(Figure 1). Collectively, the available evidence and our results suggest that crizotinib could improve the early treatment response but not benefit the overall event‐free survival. As a second‐generation of ALK inhibitor, alectinib has been characterized of favorable efficacy, higher CNS penetrating activity and better safety than crizotinib. Impressively, in a phase II study involving alectinib in treating adults with relapsed or refractory ALK‐positive ALCL, the treatment outcome is comparable to these newly‐diagnosed ALCL patients.^29^ Early relapse, occurring within 12 months after diagnosis, has been generally accepted as an independent indicator of poor outcome for ALCL. An 100% ORR was achieved in alectinib‐included therapy (Figure 1), as compared to a CR rate of 95% in ALCL patients reported on ALCL 99‐vinblastine study.^7^ Meanwhile, we observed a fast CR response fashion in alectinib‐treated patients (Table 1, and Figure 1). Furthermore, the median duration of RFS and OS was 24‐month and 100% respectively in alectinib‐included therapy. Strikingly, none of these four alectinib‐treated patients had disease progression or relapse, suggesting the potential role of alectinib in preventing early relapse. Currently, only nine ALCL cases with bronchial involvement were reported,^30^, ^31^, ^32^, ^33^, ^34^, ^35^, ^36^, ^37^, ^38^ with rapid disease progression. Introducing effective agents to treat is critical for restraining disease progression and improving their outcome. Of note, our study has shown that alectinib‐included therapy had higher anti‐ALCL activities in the patient with bronchial involvement (Figure 1D), suggesting a druggable concentration in bronchial tumor tissues.

To our knowledge, this is the first report on introducing alectinib in children with newly diagnosed ALK‐positive ALCL. Despite these promising results, our study definitely had its own limitations which could not be neglected. First, we did not perform inference testing to determine if alectinib is superior to crizotinib on patient outcomes, as the small sample size in our study may likely be under power for the testing, thus the ascertainment of alectinib superiority to crizotinib could not be conclusively drawn. Second, it is a single institutional data, a non‐randomized study, and a relative short follow‐up duration.

## AUTHOR CONTRIBUTIONS

**Yingyi He:** Conceptualization (equal); data curation (equal); formal analysis (equal); methodology (equal); writing – original draft (lead); writing – review and editing (lead). **Kunlin Pei:** Data curation (equal); formal analysis (equal); methodology (lead); writing – review and editing (supporting). **Hui Zhang:** Conceptualization (equal); data curation (equal); formal analysis (equal); funding acquisition (lead); methodology (equal); writing – original draft (equal); writing – review and editing (equal). **Jiayi Wang:** Data curation (supporting). **Xiaoling Su:** Data curation (supporting). **Wenting Gan:** Data curation (supporting). **Pengfei Wang:** Data curation (supporting).

## CONFLICT OF INTEREST

The authors had no potential conflicts of interest to disclose. All the authors have critically reviewed the manuscript and approved the submission.

## ETHICAL APPROVAL STATEMENT

This was a retrospective cohort study. Ethical approval was obtained from the ethics committee at Guangzhou Women and Children's Medical Centre (2020–136). Informed consent was provided by the patients' legal guardians, or patients themselves if they were over 8 years old.

## Supporting information


Data S1

Figures S1‐S2

## Data Availability

Not available.
