# Supplementary material for: Observation of Alectinib‐ and Crizotinib‐ included chemotherapy in children with ALK‐positive anaplastic large cell lymphoma: A single institutional experience
Source: Cancer Med. 2022 Nov 21;12(6):7182–8. doi: 10.1002/cam4.5479 (PMC10067055; doi:10.1002/cam4.5479)
Supplement: Supplementary file 1 — Data S1 [file CAM4-12-7182-s002.docx]

**Supporting Information**

**Materials and Methods**

**Patients**

This was a retrospective cohort study. Ethical approval was obtained from the ethics committee at Guangzhou Women and Children’s Medical Centre (2020-136). Informed consent was provided by the patients’ legal guardians, or patients themselves if they were over 8 years old. Medical data were reviewed for newly patients treated with ALK inhibitors during February 2017 and September 2021. Patients who met the following inclusion criteria were enrolled for further analysis: 1) age < 18 years; 2) newly diagnosed stage III or IV ALK-positive ALCL; 3) introduction of ALK inhibitor during therapy; 4) the Eastern Cooperative Oncology Group (ECOG) performance status of 0 - 2; and 5) patient had at least one measurable lesion^1, 2^, and preserved organ functions. Patient were excluded if they had received prior radiotherapy or a severe hypersensitivity/allergy history.

**Treatment regimens**

All patients were treated with multiagent induction chemotherapy following the consecutive EICNHL ALCL-99 study protocol^3^, concomitantly with alectinib or crizotinib as shown in **Supplementary Figure 1**. Briefly, The ALCL-99 protocol consists of a 5-day pre-phase and six following courses (A and B) every 21 days, and the duration of treatment depends on risk and stage stratification^4^. The dosage of oral alectinib was 150 mg and 300 mg twice daily for patient weighted < 25 kg, and ≥ 25 kg, respectively. Oral crizotinib was dosed at 125 mg, and 250 mg twice daily for the patient weighed <25 kg and ≥25 kg, respectively. One cycle of ALK inhibitor treatment was 21 days. ALK inhibitor treatment was continued for at least one year if patient did not have progressive disease or experienced any intolerable drug toxicity. From the second year onwards, ALK inhibitor was continued for patients who may benefit from further treatment for a maximum of 35 cycles at the treating physician’s discretion upon assessment. After achieving complete remission, patient may proceed to auto-SCT if this therapy was regarded largely benefiting their favorable outcome.

In addition, patient 1, 3, 6 and 7 were treated with CHOP regimens (Cyclophosphamide 750 mg/m^2^, Dexamethasone 10mg/m^2^, Doxorubicin 40 mg/m^2^, Vinblastine 1.5mg/m^2^) in prior with suspected lymphoma under severe life-threaten status. Patient 4 experienced hemophagocytic lymphohistiocytosis (HLH) before the ALCL diagnosis, and then was treated with two doses of etoposide (150 mg/m^2^/dose, every week) and ten days of dexamethasone (10 mg/m^2/^day). In the meanwhile, patient 2 and 5 were treated strictly following the ALCL-99 protocol.

**Response assessment and outcomes**

Response evaluation were performed using the international pediatric non-Hodgkin lymphoma response criteria (IPNHLSS)^2, 5^ from the 4^th^ week of chemotherapy, on 2 separate assessments in 4 weeks or longer apart using computed tomography (CT) scan. Since 18F-fluorodeoxyglucose (FDG) positron-emission tomography (PET)/CT is recommended in the earlier Cheson criteria, all patients underwent the (PET)/CT modality at the 1-year follow up. TCR rearrangement analysis was determined using multiple PCR. Flow cytometry for minimal residual disease (MRD) testing had a sensitivity of 0.01% or higher. Evaluation was also carried out for all patients receiving ALK inhibitor as a single agent when chemo treatment was complete.

Relapse-free survival (RFS) was determined from the date of achievement of response to relapse, or death, or censoring at the last follow-up. Event-free survival (EFS) was calculated from date of relapse to last follow-up or first event (secondary relapse, secondary malignancy, or death from any cause). Overall response rate (ORR) was defined as the proportion of patients who achieved complete remission (CR) or partial remission (PR). Over survival (OS) was determined from the date therapy started to the date of death or censoring at the last follow-up.

**Quantification of *NPM*-*ALK* transcripts**

Bone marrow (BM) samples from two BM involvement ALCL patients (patient 1 and patient 7) were preserved in the tissue bank. Quantitative real-time PCR (qRT-PCR) was used to monitor the BM *NPM*-*ALK* transcript using bone marrow samples before and after treatment. Bone marrow mononuclear cells were separated using Ficoll pague density gradient isolation. Total RNA was extracted using the RNeasy Micro kit (Qiagen) according to the manufacturer’s protocol and then reversely transcribed into cDNA. The qRT-PCR was performed with Faststart SYBR Green master mix (Roche)using an ABI Prism 7900HT detection system (Applied Biosystems). GAPDH was used as an internal control. Sequences of primers were listed as below: *NPM-ALK* forward: 5’-GGGCCAGTGCATATTAGTGGA-3’, reverse: 5’-AGTGTACCGCCGGAAGCACC-3’; and Probe FAM- TGTACTCAGGGCTCTGCAGCTAGCACTTAGT - TAMRA.

**Evaluation of the side-effects**

Side-effect was defined as an unrelated pharmacological effect occurring within the ALK inhibitor application with or without systemic chemotherapy. Adverse events were graded according to the Common Terminology Criteria for Adverse Events version 5.0.

**Clinical follow up**

The clinical follow-up was performed using the scheme published by Alfred Reiter et al^3^, with every 2 to 4 months for the first 3 years, every 6 months during years 4 and 5, and then yearly. Relapse was confirmed by radiological tests and biopsy.

**Statistical analysis**

Data were pooled for analysis, and descriptive statistics were used to summarize the data, using SAS® Version 9.2 or higher.

**Supplementary Figure Legends**

**Supplementary Figure 1.** **Individual treatment schema in this study.** The timeline plots the treatment schema in these seven patients (red arrow line, ALK inhibitor; green box, CHOP therapy; blue box, ALCL-99 protocol; purple box, HLH04 therapy).

**Supplementary Figure 2. Representative CT scan images of patient 3 before (A) and after (B) alectinib-included ALCL therapy.**

**REFERENCES**

1. Cheson BD, Fisher RI, Barrington SF, Cavalli F, Schwartz LH, Zucca E, et al. Recommendations for initial evaluation, staging, and response assessment of hodgkin and non-hodgkin lymphoma: The lugano classification. J Clin Oncol 2014; 32:3059-68.

2. Sandlund JT, Guillerman RP, Perkins SL, Pinkerton CR, Rosolen A, Patte C, et al. International pediatric non-hodgkin lymphoma response criteria. J Clin Oncol 2015; 33:2106-11.

3. Brugieres L, Le Deley MC, Rosolen A, Williams D, Horibe K, Wrobel G, et al. Impact of the methotrexate administration dose on the need for intrathecal treatment in children and adolescents with anaplastic large-cell lymphoma: Results of a randomized trial of the eicnhl group. J Clin Oncol 2009; 27:897-903.

4. Wrobel G, Mauguen A, Rosolen A, Reiter A, Williams D, Horibe K, et al. Safety assessment of intensive induction therapy in childhood anaplastic large cell lymphoma: Report of the alcl99 randomised trial. Pediatric blood & cancer 2011; 56:1071-7.

5. Rosolen A, Perkins SL, Pinkerton CR, Guillerman RP, Sandlund JT, Patte C, et al. Revised international pediatric non-hodgkin lymphoma staging system. J Clin Oncol 2015; 33:2112-8.
