# Supplementary material for: Observation of Alectinib‐ and Crizotinib‐ included chemotherapy in children with ALK‐positive anaplastic large cell lymphoma: A single institutional experience
Source: Cancer Med. 2022 Nov 21;12(6):7182–8. doi: 10.1002/cam4.5479 (PMC10067055; doi:10.1002/cam4.5479)
Supplement: Supplementary file 2 — Figures S1‐S2 [file CAM4-12-7182-s001.pptx]

## Slide 1
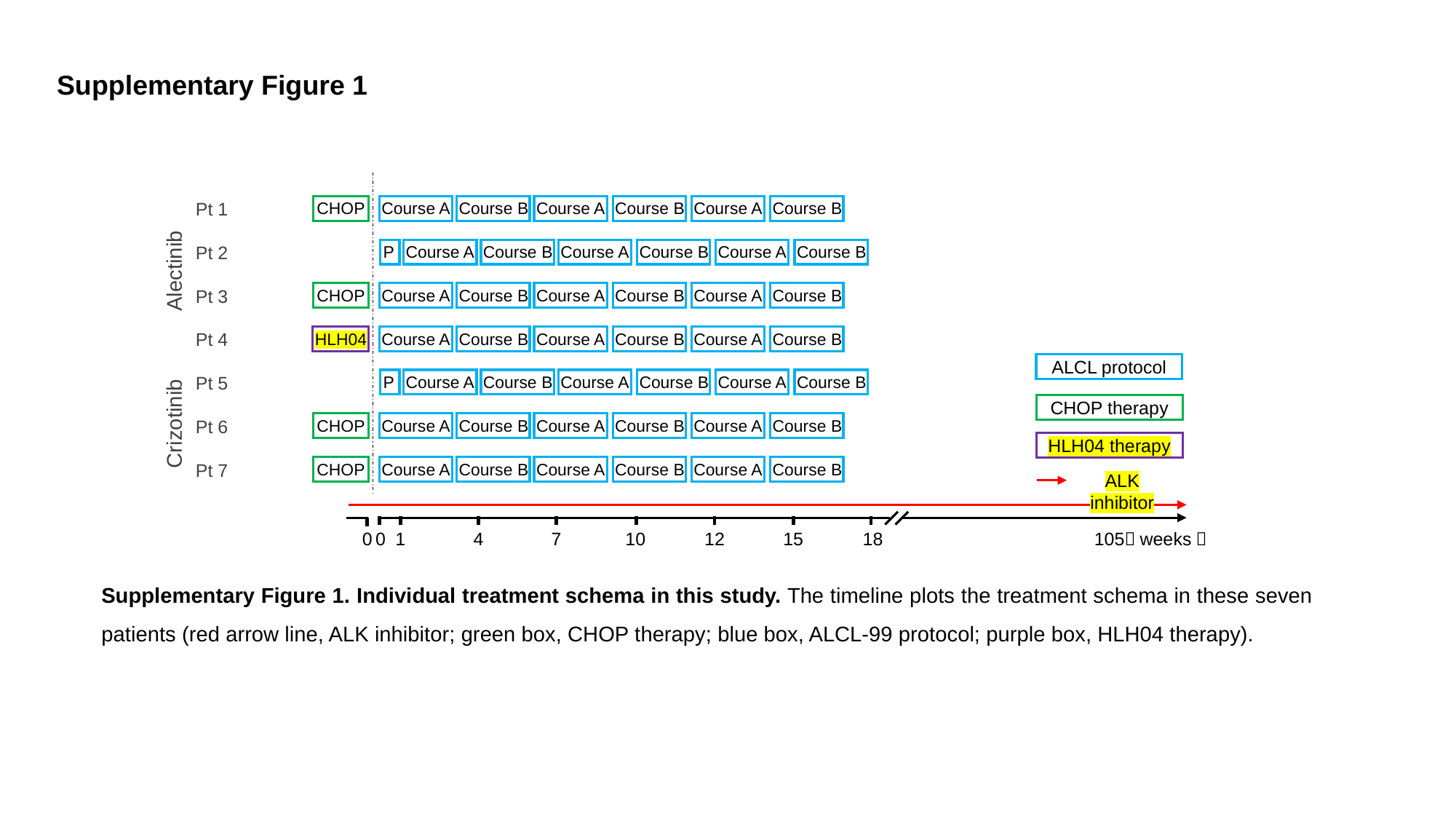

Supplementary Figure 1
Pt 1
CHOP
Course A
Course B
Course A
Course B
Course A
Course B
Pt 2
P
Course A
Course B
Course A
Course B
Course A
Course B
Alectinib
Pt 3
CHOP
Course A
Course B
Course A
Course B
Course A
Course B
Pt 4
Course A
Course B
Course A
Course B
Course A
Course B
HLH04
ALCL protocol
Pt 5
P
Course A
Course B
Course A
Course B
Course A
Course B
CHOP therapy
Pt 6
CHOP
Course A
Course B
Course A
Course B
Course A
Course B
Crizotinib
HLH04 therapy
Pt 7
CHOP
Course A
Course B
Course A
Course B
Course A
Course B
ALK inhibitor
0
0
1
4
7
10
12
15
18
105
（weeks）
Supplementary Figure 1. Individual treatment schema in this study. The timeline plots the treatment schema in these seven patients (red arrow line, ALK inhibitor; green box, CHOP therapy; blue box, ALCL-99 protocol; purple box, HLH04 therapy).

## Slide 2
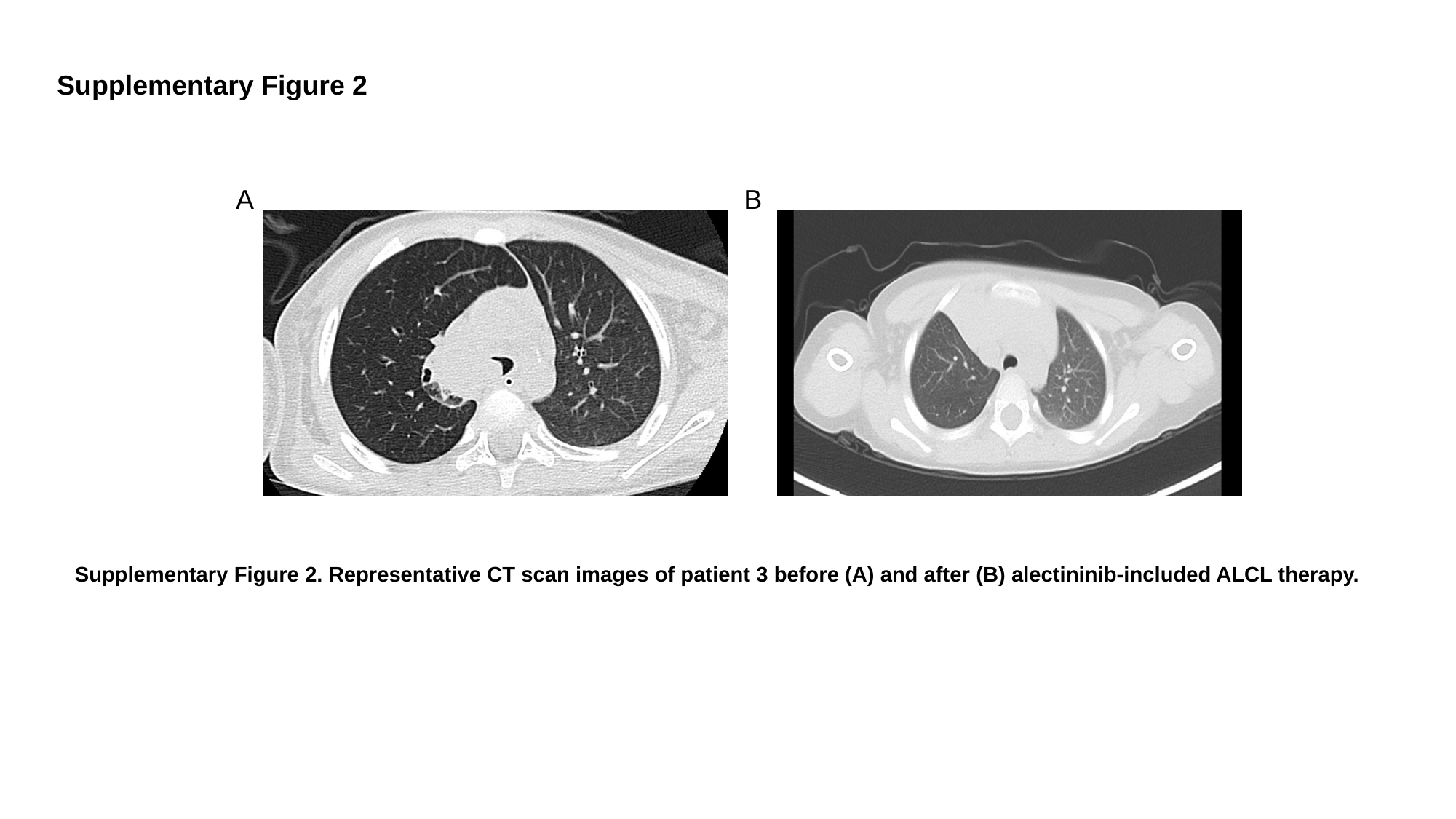

Supplementary Figure 2
A
B
Supplementary Figure 2. Representative CT scan images of patient 3 before (A) and after (B) alectininib-included ALCL therapy.
